# Banxia Baizhu Tianma decoction for hyperlipidemia: Protocol for a systematic review and meta-analysis

**DOI:** 10.1097/MD.0000000000013067

**Published:** 2018-11-02

**Authors:** Hairong Cai, Yongning Guo, Zicong Zhao, Yanhong Chen, Shuai Zhao, Bojun Chen

**Affiliations:** a The Second Clinical Medical School, Guangzhou University of Chinese Medicine; b Department of Emergency, The Second Affiliated Hospital of Guangzhou University of Chinese Medicine, Guangzhou, Guangdong Province, China.

**Keywords:** Banxia Baizhu Tianmao decoction, hyperlipidemia, protocol, systematic review

## Abstract

**Background:**

Hyperlipidemia is one of the most common metabolic diseases worldwide, as well as a leading risk factor for cardio-cerebrovascular diseases. Banxia Baizhu Tianma decoction (BBTD) is widely used for the treatment of hyperlipidemia in China, however, systematic review and meta-analysis regarding its efficacy and safety is lacking. The aim of this protocol of systematic review is to evaluate the effectiveness and safety of BBTD in the treatment of hyperlipidemia.

**Methods:**

We will include the randomized controlled trials estimating the effectiveness and safety of BBTD in the treatment of hyperlipidemia. Data sources will include 5 English databases (PubMed, EMBASE, Cochrane Library, Web of Science, CINAHL) and 4 Chinese databases (CMB, CNKI, VIP, Wanfang database). The literature to be collected will be from the time when databases were established to September 2018. Efficacy will be accepted as the primary outcome, while blood lipid levels and adverse reactions as the secondary outcome. Study selection, data collection, risk of bias assessment, and evaluation of the quality of the evidence will be conducted by 2 × 2 different reviewers. Statistical syntheses will be conducted by using RevMan software V.5.3.

**Results:**

This study will provide a high-quality evidence for BBTD on the treatment of hyperlipidemia from efficacy, serum lipid levels, and adverse reactions.

**Conclusion:**

The result will provide a basis for judging whether BBTD is an effective intervention for the treatment of hyperlipidemia.

**PROSPERO registration number:**

PROSPERO CRD 42018081359.

## Abstract 2

Supplemental Digital Content is available in the text

## 1 Introduction

Hyperlipidemia (HL) also called as dyslipidemia,^[1]^ is a one of the most common metabolic disease among middle-aged and elderly people. Its clinical manifestations include hypercholesterolemia, hypertriglyceridemia, mixed hyperlipidemia, and low-density lipoprotein cholesterol. A large number of epidemiological investigations have shown that hyperlipidemia is a leading risk factor for many diseases such as atherosclerosis, hypertension, coronary heart disease, diabetes, and stroke,^[2–5]^ which will not only lead to high disability and fatality, but also a large amount of medical and social resources, and heavy burden on family and national health care. For every 1 mmol/L increase in cholesterol in Asians, the risk of death due to cardiovascular disease increases by 35%, and the risk of blood vessel-related stroke increases by 25%.^[6]^ The CTT meta-analysis showed that for every 1.0 mmol/L reduction in low-density lipoprotein cholesterol (LDL-C), the risk of vascular disease was reduced by 21% and the risk of vascular disease death reduced by 12%.^[7]^ In China, the prevalence of dyslipidemia in adults aged 18 and over is 18.6%, 22.2% in men, and 15.9% in women.^[8]^ Statins is the main treatment adopted for lowering the levels of total cholesterol (TC) and LDL-C by inhibiting 3-hydroxy-3-methylglutaryl coenzyme A (HMG-CoA). It is widely used as the most commonly prescribed pharmacological agents for hyperlipidemia and secondary prevention of coronary heart disease.^[9]^ However, the clinical application of stains is restricted to some extent due to adverse reactions, such as elevated levels of transaminases, rhabdomyolysis, new-onset of diabetes, and intolerance.^[10]^ Traditional Chinese Medicine (TCM), an important part of complementary and alternative medicine (CAM), are widely accepted and used in clinical practice.^[11]^ A large number of studies have shown that there is good curative effect for Chinese medicine and acupuncture in the treatment of hyperlipidemia.^[12]^ Banxia Baizhu Tianma decoction (BBTD) is made up of 6 kinds of TCM: Banxia (*Pinellia tuber*), Tianma (*Gastrodia elata*), Fuling (Indian bread), Juhong (Citrusmaxima), Baizhu (*Atractylodes macrocephala*), Gancao (Liquorice root), all of which are standardly marked in Chinese Pharmacopoeia (V.2015). An animal experiments have shown that BBTD could reduce serum TC, triglyceride (TG), LDL-C, apolipoprotein B (apoB), superoxide dismutase (SOD), malondialdehyde (MDA) in rats with hyperlipidemia.^[13]^ Wang^[14]^ and He et al^[15]^ found that BBTD was effective for the treatment of hyperlipidemia, however, there is a lack of systematic review and meta-analysis regarding its efficacy and safety in the treatment of HL. Therefore, this systematic review will evaluate whether BBTD is effective and safe in the treatment of HL, in order to provide a stronger evidence-based medical basis for clinical application.

## 2 Methods

### 2.1 Inclusion criteria for study selection

#### 2.1.1 Types of studies

All randomized controlled clinical trials (RCTs) of BBTD for the management of patients with HL, whether blinded or not, will be included. There will be no restrictions on methodological quality of eligible RCTs, language, or time.

#### 2.1.2 Types of patients

Participants, adult patients (18 years of age and older) with HL whose blood lipids are abnormal for 2 consecutive tests after stoping lipid regulating agent and diet therapy 2 to 4 weeks later will be include. HL should be confirmed according to the standard diagnostic criteria including the “Guidelines for the prevention and treatment of dyslipidemia in Chinese adults (2016),”^[16]^ Executive summary of the third report of the National Cholesterol Education Program (NCEP) expert panel on detection, evaluation, and treatment of high blood cholesterol in adults (Adult Treatment Panel III).^[17]^ There will be no restrictions on age, sex, race, nationality, and comorbidity. We will exclude animal studies and trials that are primarily conducted in children (17 years of age and younger).

#### 2.1.3 Types of interventions

The therapy used in the experimental group will be BBTD by oral administration including decoction, granules, or TCM with other modern dosage forms. The interventions used in the control group include placebo, blank control, and conventional medicine (such as stains). If combined treatment of BBTD and conventional pharmacotherapy were used in the experimental group. The same conventional pharmacotherapy must be used in the control group. The administration time of each group is not <4 weeks.

#### 2.1.4 Types of outcome measures

##### 2.1.4.1 Primary outcomes

The primary outcome indicators are determined according to the Guiding Principles for Clinical Research of Drugs^[18]^: markedly effective: TC decreased ≥20% or TG decreased ≥40% or HDL-C increased ≥0.26 mmol/L; 2 effective: TC decreased by 10% to 20% or TG decreased by 20% to 40% or HDL-C increased by 0.10 to 0.26 mmol/L; 3 invalid: did not meet the above criteria.

##### 2.1.4.2 Secondary outcomes

The secondary outcome includes Serum TC, TG, LDL-C, HDL-C, apolipoprotein A (apo A), apo B, adverse reactions (nausea, vomiting, diarrhea, and so on).

### 2.2 Search methods for the identification of studies

Five English databases (PubMed, EMBASE, Cochrane Library, Web of Science, CINAHL) and 4 Chinese databases (CMB, CNKI, Chinese Science and Technology Periodical database [VIP], Wanfang database) will be searched from inception to September 2018 for the relevant RCTs of BBTD for HL. Search terms will be as follows: HL, BBTD, and RCTs. The strategy for searching the PubMed will be shown as an example in Appendix A (Supplemental Appendix A), and modified by using other databases.

#### 2.2.1 Searching other resources

We will manually search for references that have been included in relevant literature or systematic review, specialist journals, and conference proceedings. We will also use Google Scholar and other search engines to find relevant documents on the Internet. Furthermore, we will contact experts in the field to see if they understand other research topics. Additional trials included WHO Trial Register, ClinicalTrials.gov42 will be attained.

### 2.3 Data collection and analysis

#### 2.3.1 Selection of studies

**Figure 1:**
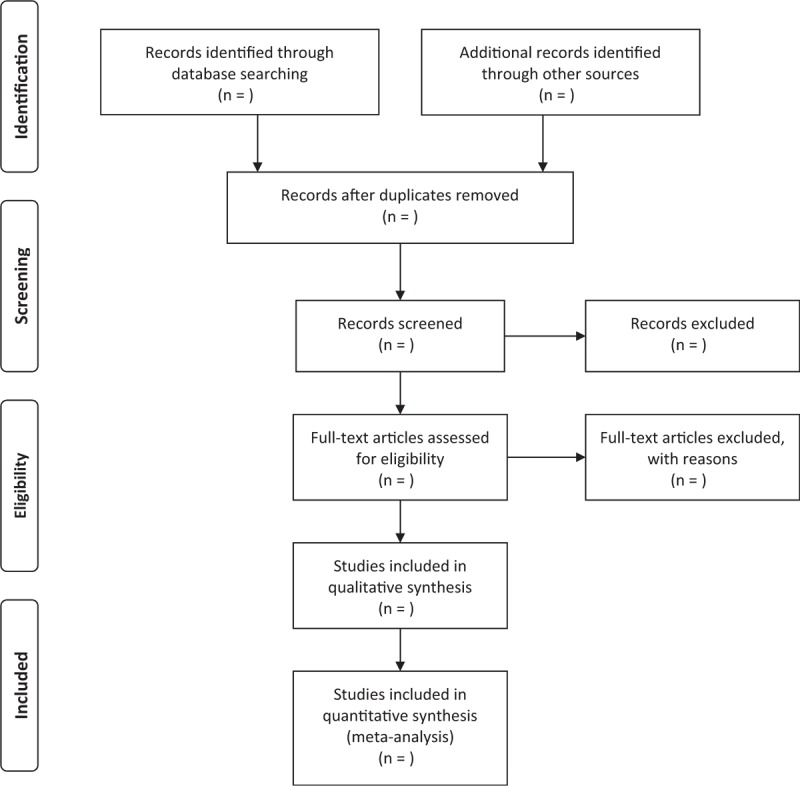
Preferred reporting items for systematic review and meta-analysis (PRISMA) flow chart.

The retrieved articles will be imported into the document management system of EndNote software (Version 9.0, Connecticut: Thomson ResearchSoft, 2018), which automatically eliminate the duplicatd articles by the corresponding researchers; later, 2 independent review authors will read the titles and abstracts to exclude the obvious disqualified literatures according to the pre-established inclusion and exclusion criteria. Then, another 2 review authors will read the full text of the literature to determine whether it meet the inclusion criteria or not. When there are different opinions, they can reach consensus by mutual discussion and by consulting a third author. Eventually, another review author will check the final included literature. The process of studies selection and meta-analysis is presented in an adapted preferred reporting items for systematic review and meta-analysis (PRISMA) flow diagram (Fig. 1).

#### 2.3.2 Data extraction and management

The information will be extracted by a standardized data abstraction form, including basic research information, research methods, observational conditions, intervention and control measures, measurement indicators, results, and adverse reactions, which will be carried out independently by 2 authors. Any disagreement will be resolved by discussion and consulting a third author. The authors will contact the author for further information, if the information of the articles is incomplete. The third review author will check the results of the extraction.

#### 2.3.3 Assessment of risk of bias in included studies

The quality and risks of the included literature will be evaluated by 2 independent review author will according to the Risk of bias tool recommended by the Cochrane Handbook V.5.1, including random sequence generation, random allocation concealment, subject and researcher blind method implementation, outcome reviewer blind method implementation, result data integrity, selective outcome report, and other biases. The quality will be low bias risk, high bias risk, and uncertain. A database of evaluation forms was established using Microsoft Excel software (Microsoft Office 2016, Redmond: Microsoft Corporation, 2016) and literature quality evaluation information was entered and analyzed. The third review author will check the result. Any inconsistencies will be resolved by discussion and consulting a third author.

#### 2.3.4 Measures of treatment effect

The relative data (RR) with 95% confidence interval (CI) will be used to evaluate enumeration data, and standardized mean difference (SMD) with 95% confidence interval (CI) for continuous data. *P* < .05 was considered statistically significant.

#### 2.3.5 Dealing with missing data

If required data in the included literature is disappeared or unclear, the author will contact the first or corresponding author by e-mail to obtain complete information. If no additional information are received, we will use the available data for data synthesis. At the same time, we will also discuss the potential impact of the missing data in the discussion.

#### 2.3.6 Assessment of heterogeneity

Heterogeneity of the result will be analyzed by *X*^2^ test (*a* = 0.1) and expressed as *I*^2^ value. If *I*^2^ < 25%, the heterogeneity was small, >25% and <50%, moderate, and *I*^2^ > 50%, the heterogeneity is large. If the *I*^2^ value exceeds 50%, a subgroup analysis will be performed to investigate the potential causes from clinical or methodological heterogeneity.

#### 2.3.7 Assessment of reporting bias

The publication bias will be evaluated by the visual asymmetry on a funnel plot, if at least 10 trials are included in the study.

#### 2.3.8 Data synthesis

Data synthesis will be performed by using RevMan software (Version 5.3, Copenhagen: The Nordic Cochrane Center, 2014) provided by the Cochrane Collaboration. If there is statistical hemogeneity between the results (*P* > .1, *I*^2^ < 50%), the fixed-effects model will be conducted for meta-analysis; if not (*P* ≤ .1, *I*^2^ ≥ 50%), subgroup analysis will be performed to investigate the sources of heterogeneity. If the result of subgroup analysis shows that there is sufficient similarity between the subgroups (*P* > .1, *I*^2^ < 50%), the fixed-effect model will be used for meta-analysis; otherwise, if there is statistical heterogeneity but no clinical heterogeneity between the subgroups, the random-effects model will be conducted. The subgroup or sensitivity analysis, or only descriptive analysis will be performed if there is obvious clinical heterogeneity.

#### 2.3.9 Subgroup analysis

Subgroup analysis will be performed according to different interventions, participants, sex, duration of disease, and dose of medication to explore the source of heterogeneity if the included studies are sufficient (at least 10 trials).

#### 2.3.10 Sensitivity analysis

Sensitivity analysis according to sample size, missing data results, and methodological quality will be performed to identify the quality.

#### 2.3.11 Grading the quality of evidence

The quality level of evidence will be evaluated by the GRADE profiler software (Version 3.6, The GRADE Working Group, 2010). The results will be divided into 4 levels: high, medium, low, or very low, and the recommended level will be made according to the research topic.

## 3 Discussion

HL is one of the most common metabolic diseases, as well as a leading risk factor for cardio-cerebrovascular diseases. Statins are routine choices for the treatment of HL, however, the clinical application is restricted to some extent due to adverse reactions, such as elevated levels of transaminases, rhabdomyolysis, new-onset of diabetes, and intolerance. Studies have shown that BBTD could improve symptoms such as dizziness and headache, and reduce blood lipid levels in patients with HL. However, systematic review and meta-analysis regarding its efficacy and safety in the treatment of HL is lacking. Therefore, a high quality systematic review and meta-analysis is necessary and the process can be shown in the flow chart (Fig. 1). It is expected that the review will provide more convincing evidence to prove the advantages of BBTD in the treatment of hyperlipidemia. However, there may be some limitations in this review. First, the included trails are limited to Chinese or English publications, which may result in selection bias and small samples of the article may lead to high risks of bias. Second, different doses of herbs, patient age may cause a great heterogeneity risk.

## Author contributions

BC is the guarantor of the article. The manuscript was drafted by HC. The search strategy was performed by YG. ZZ and SZ will independently screen the articles, extract data, assess the risk of bias, and performed data synthesis. YC will check the final included literature and results of the extraction and bias. BC will arbitrate any disagreement and ensure that no errors occur during the review.

**Conceptualization:** Yongnig Guo, Zicong Zhao, Shuai Zhao, Yanhong Chen.

**Investigation:** Bojun Chen.

**Writing – original draft:** Hairong Cai.

**Writing – review & editing:** Hairong Cai.

## Supplementary Material

Supplemental Digital Content
