# Supplementary material for: Banxia Baizhu Tianma decoction for hyperlipidemia: Protocol for a systematic review and meta-analysis
Source: Medicine (Baltimore). 2018 Nov 2;97(44):e13067. doi: 10.1097/MD.0000000000013067 (PMC6221624; doi:10.1097/MD.0000000000013067)
Supplement: Supplemental Digital Content [file medi-97-e13067-s001.docx]

**Appendix A.**

***Search strategy used in PubMed database***

#1 hyperlipemia OR hyperlipemias OR hyperlipidemia OR lpidemia OR hipidemias OR lipemia OR lipemias OR dyslipidemia OR dyslipoproteinemias OR Dyslipoproteinemia OR lipoprotein disorder

#2 banxia baizhu tianma decoction OR ban xia bai zhu tian ma decoction OR banxia baizhu tianma tang OR banxia baizhu tianma yin

#3 Randomized controlled trial OR clinical study OR Clin-ical Trial OR Controlled study OR Controlled Trial OR Random*Control* study OR random* Control* Trial

#1 AND #2 AND #3
